# proGenomes: a resource for consistent functional and taxonomic annotations of prokaryotic genomes

**DOI:** 10.1093/nar/gkw989

**Published:** 2016-10-24

**Authors:** Daniel R. Mende, Ivica Letunic, Jaime Huerta-Cepas, Simone S. Li, Kristoffer Forslund, Shinichi Sunagawa, Peer Bork

**Affiliations:** 1 Structural and Computational Biology Unit, European Molecular Biology Laboratory, 69117 Heidelberg, Germany; 2 Daniel K. Inouye Center for Microbial Oceanography Research and Education, University of Hawai'i at Manoa, Honolulu, HI 96822, USA; 3 Biobyte solutions GmbH, Bothestrasse 142, 69126 Heidelberg, Germany; 4 School of Biotechnology and Biomolecular Sciences, University of New South Wales, 2052 Sydney, Australia; 5 Institute of Microbiology, Department of Biology, ETH Zurich, Vladimir-Prelog-Weg 4, 8093 Zurich, Switzerland; 6 Molecular Medicine Partnership Unit, University of Heidelberg and European Molecular Biology Laboratory, 69120 Heidelberg, Germany; 7 Max Delbrück Centre for Molecular Medicine, 13125 Berlin, Germany; 8 Department of Bioinformatics, Biocenter, University of Würzburg, 97074 Würzburg, Germany

## Abstract

The availability of microbial genomes has opened many new avenues of research within microbiology. This has been driven primarily by comparative genomics approaches, which rely on accurate and consistent characterization of genomic sequences. It is nevertheless difficult to obtain consistent taxonomic and integrated functional annotations for defined prokaryotic clades. Thus, we developed proGenomes, a resource that provides user-friendly access to currently 25 038 high-quality genomes whose sequences and consistent annotations can be retrieved individually or by taxonomic clade. These genomes are assigned to 5306 consistent and accurate taxonomic species clusters based on previously established methodology. proGenomes also contains functional information for almost 80 million protein-coding genes, including a comprehensive set of general annotations and more focused annotations for carbohydrate-active enzymes and antibiotic resistance genes. Additionally, broad habitat information is provided for many genomes. All genomes and associated information can be downloaded by user-selected clade or multiple habitat-specific sets of representative genomes. We expect that the availability of high-quality genomes with comprehensive functional annotations will promote advances in clinical microbial genomics, functional evolution and other subfields of microbiology. proGenomes is available at http://progenomes.embl.de.

## INTRODUCTION

**Figure 1.**
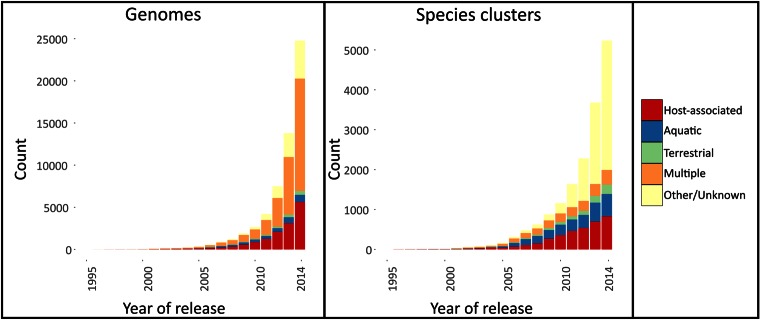
Availability of sequenced genomes and species clusters availability over time. Colors represent the habitat annotation of the genomes/species clusters.

Microbes play a major role in shaping the earth and have vast impacts on human health and well-being. Until recently, however, little was known about their diversity, genetics and functional potential. Over the last two decades, this has changed with the availability of sequenced genomes, which has revolutionized our understanding of microbes (1–3). The extensive use of genome sequencing in microbiology has led to an exponential increase in the number of sequenced bacteria and archaea (4) (Figure 1). However, a genome sequence alone, even if perfectly assembled, is of limited value without annotation to reveal interpretable information. The most basic annotation level should provide the taxonomic designation of a genome and the sequences of genes it encodes. Functional annotation of the latter can reveal, for example, the biochemical processes that underlie phenotypic features of a specific microbe.

The field of comparative genomics, through increasing efforts in the characterization of genomes, has led to new advances in our understanding of bacterial and archaeal life (5). Although such studies necessitate the use of consistently annotated genomes, the current state-of-the-art does not yet provide an easy entry point to obtain these. A number of publicly accessible databases provide genomes with basic and more elaborate annotations. The NCBI RefSeq database (6) provides a comprehensive sets of genomes with minimal annotations that include consistently predicted gene models. Other databases such as the DOE's Joint Genome Institute Integrated Microbial Genomes & Microbiomes (JGI IMG/M) database (7), the PATRIC (Pathosystems Resource Integration Center) database (8) and Ensembl Bacteria (9) provide additional layers of information for the deposited genomes through integration of other data sources. Yet, taxonomic annotations are usually provided by the submitter of the genome sequence. This leads to inconsistencies across different clades of the tree of life, especially at the species level, as the species definition for bacteria and archaea remains a highly debated topic among microbiologists (10). Furthermore, the taxonomic classification of prokaryotes is constantly updated, which hampers efforts to download subsets of species for a desired project. Obtaining a consistent functional ontology for a number of genomes can also be challenging as a number of functional databases exist, each of which covers distinct aspects of functional diversity (e.g. antibiotic resistance (11) or metabolic pathways (12)), and current genome resources are either incomplete or lack cross-referencing information.

**Figure 2.**
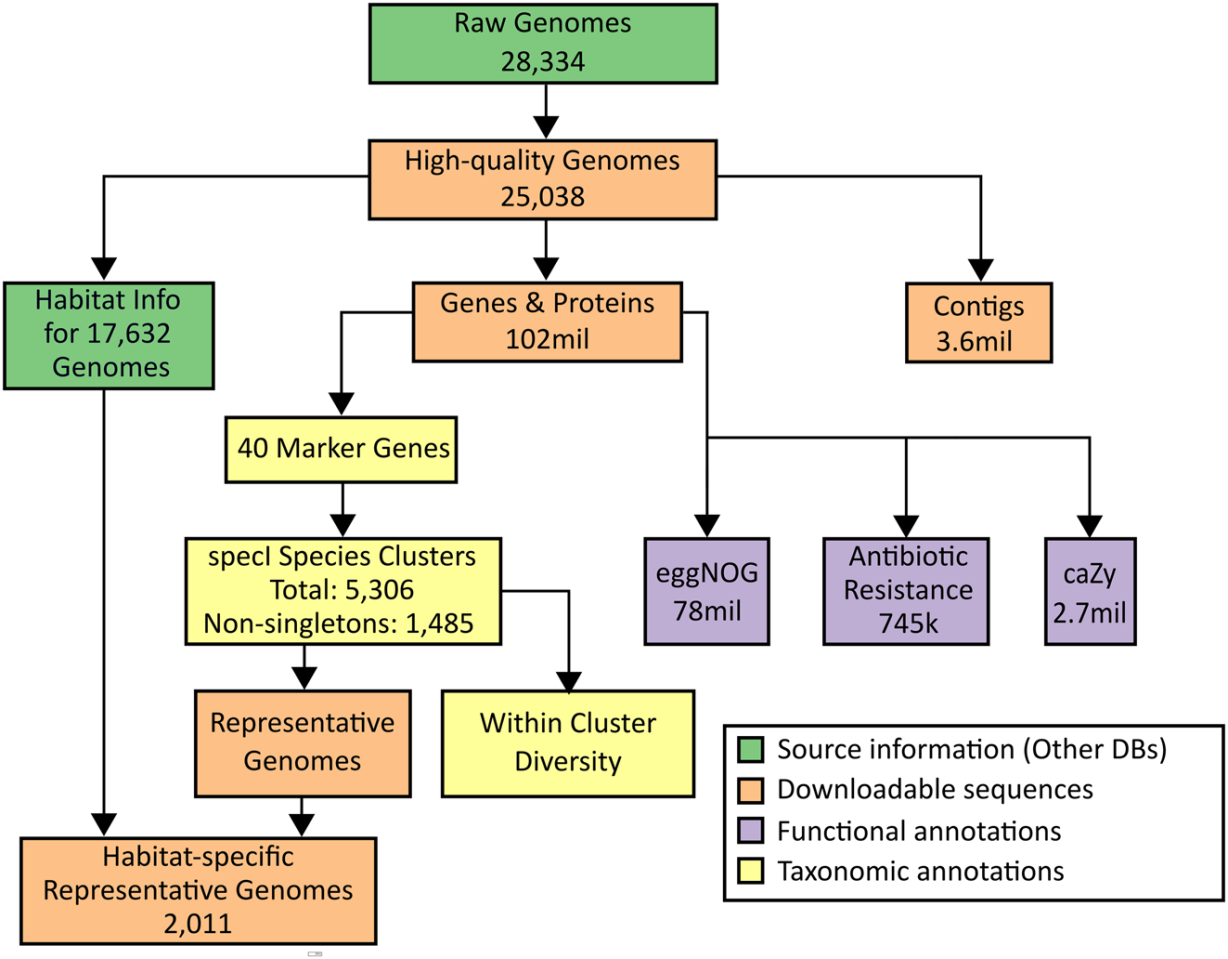
Workflow to generate the underlying data of the database.

To address these issues, we have developed proGenomes (http://progenomes.embl.de), a prokaryotic genome resource that enables direct access to genomes of any taxonomic clade, in conjunction with a number of consistently and hierarchically annotated gene functions for each genome. Additionally, we provide a robust operational species classification in the form of up-to-date species clusters, which perform well compared to the NCBI reference taxonomy (13). In order to minimize redundancy, a representative genome is selected from each specI species cluster reflecting its role in the literature and other criteria (Figure 2). The resulting non-redundant genome sets are thus well suited for metagenomic or large-scale phylogenetic studies.

The sets of genes from each genome were translated into proteomes and consistently annotated using eggNOG, one of the most comprehensive databases for orthologs and their functional annotation, with 1.9 million orthologous groups (14). Using the eggNOG-mapper tool, we were able to annotate almost 79 million protein-coding genes (15) including indirect annotations to KEGG pathways and predicted gene family names.

We also provide more specific annotations of carbohydrate-active enzymes, as well as antibiotic resistance determinants, which are additional features not currently provided together by other databases. The range of such annotations will be extended in future updates. We envision that comprehensive functional annotation of high-quality genomes will facilitate research into clinical applications of microbial genomics, as well as studies of functional evolution.

## DATABASE CONSTRUCTION AND CHARACTERISTICS

The goal of proGenomes is to provide the available microbial genomes and customizable subsets of them in a readily downloadable and user-friendly manner. Users can find genomes of interest by providing the name of a genome or a taxonomic clade. The website also allows users to explore all the provided information interactively, and genome sequences and annotations of individual genomes or whole taxonomic clades can be easily downloaded. Pre-packaged sets of representative genomes are also available for batch download. The computational pipeline that generates the data presented on the website is summarized in Figure 2. We aim to update the database up to two times per year. Further, we plan to perform major biennial updates, that will involve the integration of additional annotation sources or major improvements of existing parts of the workflow.

### Genome collection

The genome collection is based on all bacterial and archaeal genomes that were available from the NCBI Nucleotide database on 14 December 2014. Gene predictions were obtained from the deposited genomes, where available. If this information was not provided, genes were predicted using geneMarkS (16). We filtered out low quality assemblies that had an N50 score of <10k bp and/or consisted of more than 300 contigs. Incompletely assembled genomes with <30 of 40 universal, single copy marker genes were also removed (17,18). The detection of a sufficient number of these marker genes in a genome provides a universally applicable measure of genome completeness. Altogether, this resulted in a set of 25 038 high-quality genomes.

### Species clusters definitions using the specI approach

As mentioned above, the assignment of genomes to species is contentious (12). The exponentially increasing number of sequenced genomes necessitate the development and use of automatic, unbiased and systematic approaches to tackle this issue. specI species clusters provide an accurate and consistent solution, as they are based solely on genomic sequence (but also largely consistent with consensus from morphological and phenotypic evaluation) and can be applied to any set of sequenced genomes. We calculated specI species clusters using the methodology described in (13), resulted in 5306 specI species clusters for the 25 038 genomes currently in proGenome. This represents a significant advance in comparison to previous efforts, such as the MetaRef database (19), which provided a similar classification for 2818 genomes based on clade specific genes.

The specI approach utilizes a set of 40 universal, single-copy marker gene families (MGs) (17,18) that are provided as part of the resource for each genome. The MGs have been used to reconstruct the tree of life (17) and to study the phylogenetic relations within specific clades (13,20). The fetchMG tool (21) was used to extract the MGs from all high-quality genomes. To generate the updated specI species clusters, all-versus-all alignments were calculated for each of the 40 MGs using vsearch (Rognes *et al.*, https://github.com/torognes/vsearch) and genome-to-genome distances were calculated as gene length weighted mean averages. The genome-to-genome distances were then used as input for average linkage clustering. An average marker gene nucleotide identity cutoff of 96.5% was applied to generate the specI species clusters. This yielded a total of 5306 specI species clusters, of which 1485 contained more than one genome and 3821 were singletons. Non-singleton clusters contained 14.3 genomes on average. The largest cluster (specI_v2_Cluster67: Staphylococcus aureus) contained 4172 genomes. The updated specI species clusters can be easily accessed in proGenomes, either by directly searching for them or by link from any constituent genome. Due to their consistency, these clusters represent an unbiased starting point for pangenomic studies and can also serve as benchmark sets for metagenomic binning approaches.

### Selection of representative genomes

**Figure 3.**
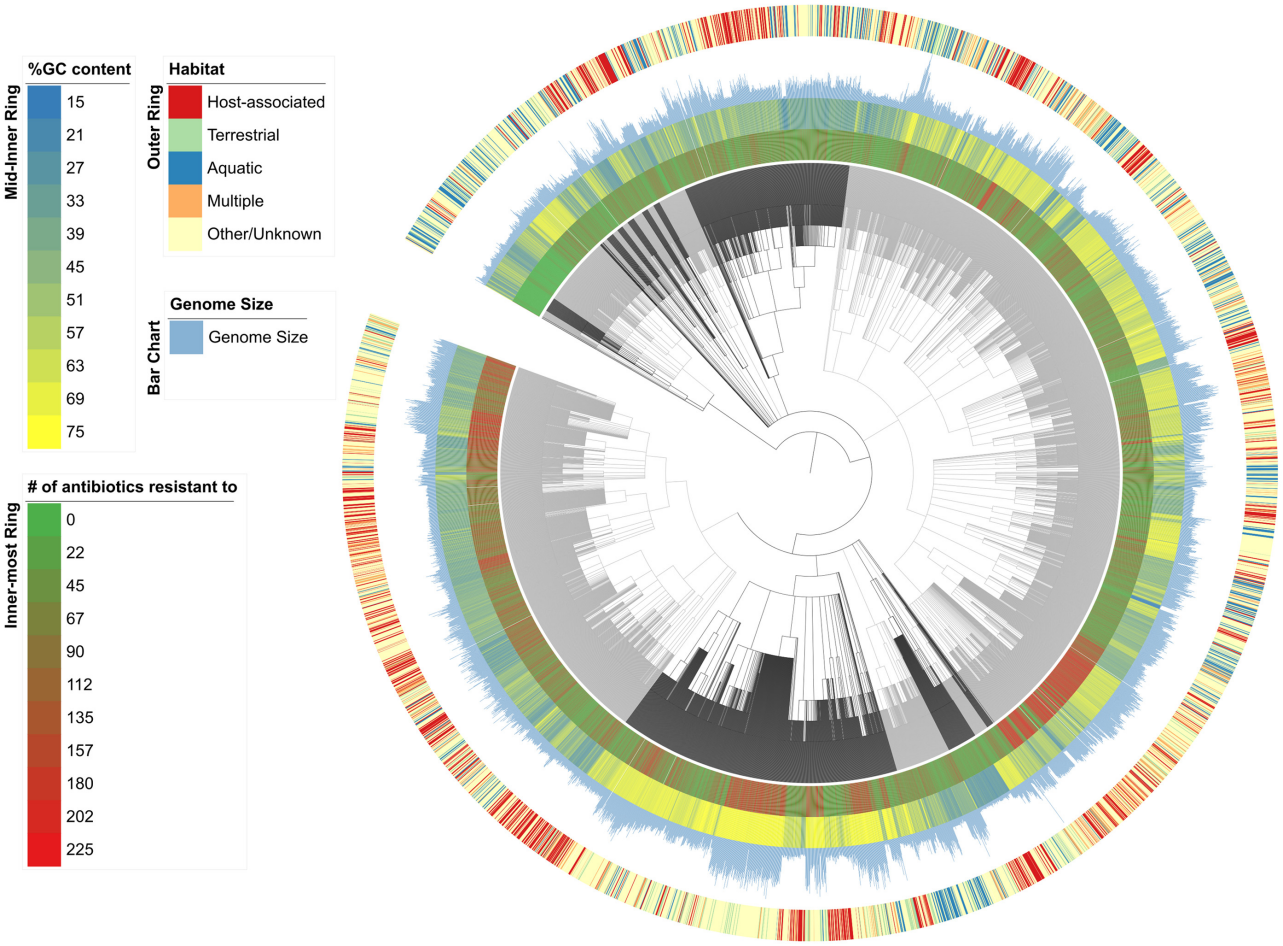
Overview of the representative genome set according to the NCBI Taxonomy. GC content, habitat information, genome size and antibiotic resistance gene carriage are displayed as additional datasets. Different Phyla are displayed as alternating light and dark gray clades within the tree (28).

Many applications in microbial genomics require non-redundant datasets. This can be due to the detrimental effects of redundancy itself (e.g. when trying to uniquely assign metagenomic reads to reference genomes, as in (22)) or because of significant efficiency gains at comparable accuracies (e.g. (14)). NCBI RefSeq currently provides a set of representative genomes from 4287 species (8), however, many species clusters were not represented in this set. We therefore provide a set of 5510 representative genomes, which are available for bulk download (Figure 3). Additionally, habitat-specific subsets of representative genomes are also available.

Before selecting the representative genomes, we established a small ‘whitelist’ of genomes of special interest. This chiefly serves to ensure iconic model organism strains are guaranteed to be shown, even where automated measures might have indicated other strains as potential representatives. Users can vote on the website for additional genomes that should be included in this whitelist in future versions of the resource.

To compile these sets of representative genomes at least one genome per specI species cluster was selected. If a specI species cluster contained one or more genomes on the whitelist these were selected. Otherwise, we selected one representative genome from every non-singleton cluster using citation statistics (reflecting the use of a strain for experimental or other model system work) as well as genome quality statistics (N50), whereby completely assembled genomes were selected preferentially. Additionally, all genomes in singleton specI species clusters were included.

### Functional annotation

The functional repertoire of a microbial genome defines its phenotype, lifestyle and ecological role. Hence, it is pivotal to our understanding of a microorganism that we have a consistent, accurate and comprehensive functional annotation of its genes. We focused on the functional annotation of protein-coding genes as they encode for most of the functional repertoire. This was achieved using the eggNOG (14) resource, as it provides a general annotation framework with a broad coverage of different protein functional categories. As mentioned above, proGenomes currently also provides focused annotations of antibiotic resistance and of carbohydrate active enzymes, with further annotations planned in future updates. Antibiotic resistance annotations are provided based on integrated results from the Comprehensive Antibiotic Resistance Database (CARD) (23) and ResFams (24) resources. For CARD, its associated resistance gene identifier tool was run on all proteins in proGenomes, with gene family assignments identified by sequence similarity using the curated CARD cutoffs and sequence (SNP) variation in antibiotic target genes identified using alignment to Hidden Markov Models (HMMs). For each proGenomes protein, the best hit above cutoff was retained in the case of resistance gene family annotation. Similarly, the set of SNPs to the best-scoring model above cutoff was retained for sequence variants. For proteins with no CARD resistance gene annotation, the best ResFams HMM hit above threshold was retained. Since both databases map to the antibiotic resistance ontology (ARO), the ARO hierarchy (as per CARD version 1.7) was used to assess which antibiotics each resistance gene determinant protects against. Proxy terms for ‘unspecified beta-lactam’ and ‘multidrug efflux pump’ were added to reconcile ambiguities in some annotations. For complexes listed in the ARO, such as components with disparate subunits, such synergies between hits were counted within each genome, reflecting how the presence of several interacting antibiotic resistance genes can provide further resistance. Carbohydrate-active enzyme annotations as defined by CAZy (25) were generated using the dbCAN HMM models (26).

Overall, almost 80 Million protein-coding genes were annotated (eggNOG: 78 921 163; CAZy: 2 704 372; CARD + ResFams: 745 070). This information can be examined interactively on the proGenomes website.

### Habitat information

Habitat information is provided for most genomes in the proGenomes database. This information can be utilized for in depth studies of selected environments or comparisons between different habitats such as (27). Habitat information was obtained from the manually curated PATRIC database (10) (accessed 15 March 2015). Specifically, the ‘Host Name’, ‘Body Sample Site’, and ‘Habitat’ fields were used. In cases where, for example, different assemblies for the same organism existed, data was collated for each NCBI Taxonomy ID. Information was available for 17 632 of the 25 038 organisms. This enabled us to broadly classify each specI species cluster into one of four different habitat types: host-associated (835), aquatic (566), terrestrial (234) or multiple (376) (Figures 1 and 3). Representative genomes for these subsets of clusters are available for bulk download from the website.

### Website

**Figure 4.**
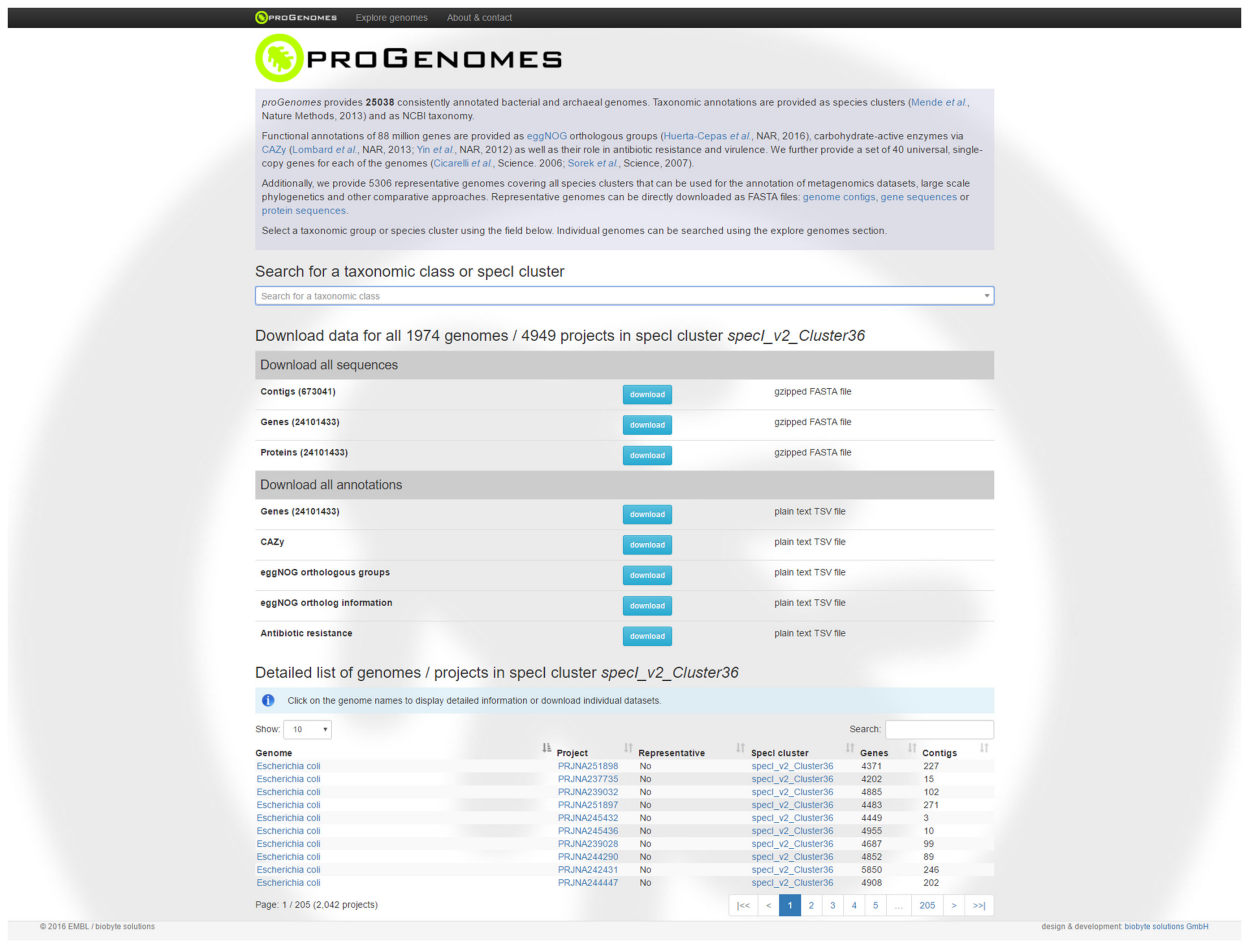
Clade/specI species cluster view on the proGenomes website. All sequences and annotations for the genomes within a clade/specI species cluster can be downloaded directly. Individual member genomes can be accessed at the bottom of the page.

The proGenomes website (http://progenomes.embl.de) can be used to browse the resource and enables direct access to the whole database. It has a searchable interface that can be used to find data from any taxonomic group or specI species cluster (Figure 4). All provided information can be explored interactively at the level of taxonomic groups or individual genomes. For larger taxonomic groups, information about all genomes within that group is displayed, with direct access to the genome, gene and protein sequences and annotations. For individual genomes, we provide all annotations in an interactive environment, which enables users to access additional information through direct links to relevant external database entries.

## DISCUSSION

proGenomes provides consistent taxonomic and functional annotations for a large number of quality filtered genomes, as well as a non-redundant, habitat-specific sets of representative genomes. The easy-to-use website provides a wide range of information relevant to researchers interested in microbial genomics and allows the customization of subsets of genomes for download, thus facilitating comparative studies that address questions from evolution, population genetics, functional genomics and many other research fields. We intend proGenomes to be a valuable resource for studies ranging from those focusing on one or a few organisms to those analyzing large-scale evolutionary patterns or complex microbial communities.
